# Bouldering psychotherapy is effective in enhancing perceived self-efficacy in people with depression: results from a multicenter randomized controlled trial

**DOI:** 10.1186/s40359-021-00627-1

**Published:** 2021-08-26

**Authors:** André Kratzer, Katharina Luttenberger, Nina Karg-Hefner, Maren Weiss, Lisa Dorscht

**Affiliations:** 1 Center for Health Services Research in Medicine, Department of Psychiatry and Psychotherapy, University Hospital Erlangen, Friedrich-Alexander University Erlangen-Nürnberg (FAU), Schwabachanlage 6, 91054 Erlangen, Germany; 2 Department of Psychology, Friedrich-Alexander University Erlangen-Nürnberg (FAU), Nägelsbachstraße 49c, 91052 Erlangen, Germany

**Keywords:** Self Efficacy, Depression, Physical Exercise, Bouldering, Therapeutic climbing, Psychotherapy

## Abstract

**Background:**

Recent studies have suggested that therapeutic climbing/bouldering may have positive effects on perceived self-efficacy. Nevertheless, there is still an urgent need for high-quality studies, as many existing studies have suffered from methodological problems. Therefore, the current work was aimed at investigating the effect of a manualized bouldering psychotherapy (BPT) on perceived self-efficacy in people with depression, compared with a home-based physical exercise program (EP) and state-of-the-art cognitive behavioral group therapy (CBT).

**Methods:**

In a prospective, multicenter, randomized controlled trial, 233 people with depression were randomly assigned to one group (BPT, EP, or CBT). Perceived self-efficacy was assessed at baseline (t0) and directly after the 10-week intervention period (t1) with the GSE. In addition, depression was assessed with the PHQ-9 and the MADRS*.* We computed *t* tests, analyses of variance (ANOVAs), confounder-adjusted hierarchical regression analyses, mediation analyses, and several sensitivity analyses.

**Results:**

BPT participants showed a significantly larger increase in perceived self-efficacy on the GSE compared with the EP (an increase of 3.04 vs. 1.26 points, *p* = .016, Cohen’s *d* = 0.39). In the confounder-adjusted hierarchical multiple regression analysis, group allocation (BPT vs. EP) was found to be the only significant predictor of the postintervention GSE score (β = .16, *p* = .014) besides the baseline GSE score (β = .69, *p* < .001). No differences were found between BPT and CBT participants regarding the effect on perceived self-efficacy. Only in the CBT group, the relationship between depression at baseline and postintervention was partially mediated (23%) by perceived self-efficacy.

**Conclusions:**

Participation in the manualized BPT in a group setting leads to a clinically relevant enhancement of perceived self-efficacy in people with depression. This effect is superior to that of physical exercise alone. The results provide also initial indications that BPT is comparable to CBT in enhancing perceived self-efficacy, suggesting a strong case for a broader use of BPT as a supplement to existing health services. Future studies should focus on the modes of action of BPT and its effect on perceived self-efficacy in people with other mental or physical disorders.

*Trial registration* ISRCTN12457760, registered partly retrospectively, 26 July 2017.

## Background

Perceived self-efficacy, defined as people’s beliefs in their capabilities to perform difficult or novel tasks to attain desired outcomes [1–3], is considered to be one important common factor of psychotherapy, not solely in the treatment of depression, but in general across all forms of disorders [4, 5]. It affects not only the initiation but also the persistence of coping behavior, especially in difficult or threatening situations [1, 6], and should therefore be addressed in psychotherapy. Enhanced perceived self-efficacy is also regarded as an important protective factor that promotes psychological resilience [2]. Furthermore, recent research also found positive effects of enhanced self-efficacy on general well-being, health behavior, pain tolerance, and active coping with difficult situations [7]. Beyond this, enhanced perceived self-efficacy is related to lower levels of depression and anxiety [7, 8].

Bouldering, i.e. climbing without ropes or harnesses at moderate heights [9, 10], is an activity that can address all of the major sources of perceived self-efficacy according to Bandura [1, 3]: *Performance accomplishments* can be quickly achieved in bouldering even by beginners because much progress and many successes can be achieved not only by gaining strength but especially by developing technique, and participants in climbing groups often have positive experiences right from the beginning [11]. Through therapeutic guidance, people can attribute such performance accomplishments to their own competencies and abilities and transfer them to everyday experiences. A group setting, recommended by Lukowski [12], also offers many opportunities for *vicarious experiences* through the observation of models who have similar psychological problems and psychosocial impairments in coping with difficult climbing routes. According to Bandura [1, 3], models should be similar to the observer and should initially have trouble coping with the situation but should constructively overcome such challenges with time through the appropriate use of functional strategies and determined effort. In addition, therapeutic climbing in a group setting also offers opportunities for *verbal persuasion* during bouldering in the form of encouragement as well as advice and support from therapists or other participants [13]. Last but not least, the possibility of opening up to, accepting, and integrating feelings in therapeutic climbing along with therapists and other participants [13] can also have a positive influence on *emotional arousal*.

Indeed, systematic reviews have confirmed that therapeutic climbing may have positive effects on health problems [14, 15] and especially on perceived self-efficacy [15], but such reviews have also highlighted the need for future high-quality studies, as many of the existing studies have suffered from methodological problems. In our randomized waitlist-controlled pilot study [16], bouldering psychotherapy (BPT)—a novel intervention combining psychotherapeutic methods with bouldering exercises—led to a significant enhancement of perceived self-efficacy in people with depression.

Based on these promising results, we decided to conduct an exploratory investigation of the specific effect of BPT on perceived self-efficacy in our current multicenter, randomized controlled trial StudyKuS. The primary aim of the StudyKuS was to investigate the effect of BPT on depression (i.e., the primary outcome of the StudyKuS) in outpatients with depression compared with evaluated treatment options, i.e. physical activity alone, in the form of a home-based physical exercise program (EP), and state-of-the-art cognitive behavioral group therapy (CBT) [17]. We were able to show that the effect of BPT on depression was superior to the effect of physical activity alone [18] and was not inferior to the effect of state-of-the-art CBT [19].

In an exploratory approach, the present work investigated whether BPT differed significantly from physical activity alone in the form of an EP or from state-of-the-art CBT regarding the effect on perceived self-efficacy (i.e., one important secondary outcome of the StudyKuS). Because perceived self-efficacy is considered to be a common factor of psychotherapy [4, 5], we examined whether the effect of BPT on depression, which was already found by Karg et al. [18] and Luttenberger et al. [19], would be mediated by perceived self-efficacy in any group (BPT, CBT, and EP).

## Methods

### Study design

The present study is based on data from the StudyKuS between 2017 and 2018 [17]. The StudyKuS was a prospective, longitudinal, multicenter, randomized controlled trial conducted in three different regions (study centers) in Germany. In each region, all interventions were conducted at the same time in consecutive waves. In each region and wave, participants were randomly assigned to one of three groups: bouldering psychotherapy (BPT), a home-based physical exercise program (EP), or state-of-the-art cognitive behavioral group therapy (CBT). The intervention period lasted 10 weeks in each group. Data were collected at baseline (t0), and directly after the 10-week intervention period (t1), as well as 3 months (t2), 6 months (t3), and 12 months (t4) after the end of the intervention period. Participants in the EP and CBT groups could participate in an additional BPT intervention after the intervention period.

All procedures were approved by the Ethics Committee of the Friedrich-Alexander University Erlangen-Nürnberg (Ref. 360_16 B). Participation was voluntary, and participants were free to leave the study at any time without disadvantages. The study was registered partly retrospectively on 26 July 2017 at ISRCTN (Trial identification number: ISRCTN12457760). Participants were recruited between March 2017 and February 2018. For more information regarding the study design, please see our study protocol by Dorscht et al. [17]

### Interventions

#### Bouldering psychotherapy (BPT)

Bouldering psychotherapy (BPT) combined bouldering with psychotherapeutic methods. The intervention is manualized and comprised 10 consecutive 2-h sessions once a week in the late afternoon with a group size of 10 participants. Each group was accompanied by two climbing therapists, which were experienced in bouldering and climbing and trained in the implementation of the BPT manual by two research associates from the study headquarters. All but one (M.Sc. in Health Science) had completed a Master’s degree in Psychology and were either licensed psychotherapists or psychotherapists in training.

Each session was based on the BPT manual and followed a standardized procedure of three parts: “introduction” (approximately 20 min), “action phase” (approximately 75 min), and “closure” (approximately 25 min). Each session comprised bouldering exercises, mindfulness exercises, psychoeducation, exchange between participants and transfer to daily life, body-related relaxation exercises, and free bouldering. For a detailed description of the treatment, please see the study protocol [17].

#### Cognitive behavioral group therapy (CBT)

The cognitive behavioral group therapy (CBT) was also manualized and followed the treatment plan of a cognitive behavioral group therapy. The CBT consisted of 10 consecutive 2-h sessions once a week in the late afternoon in groups composed of 10 participants each. Each session was accompanied by two therapists who were trained in the implementation of the CBT manual by two research associates from the study headquarters. All therapists were either licensed psychotherapists or psychotherapists in training.

Each session was based on the CBT manual and followed a standardized procedure of three parts: “introduction” (approximately 30 min), “main part” (approximately 60 min), and “closure” (approximately 15 min). It combined the psychoeducational parts by Schaub et al. [20] with specific exercises and elements of strategic behavioral therapy (SBT) by Sulz [21], as well as elements of a short-term concept by Hautzinger and Kischkel [22]. For a detailed description of the treatment, please see the study protocol [17].

#### Home-based physical exercise program (EP)

The home-based physical exercise program (EP) consisted of a 20-min physical training program that was carried out at home three times a week for 10 weeks and trained the same muscle groups that are used in bouldering or climbing. At the beginning of the intervention period, participants received a training manual by post, including video instructions on DVD, training material (e.g. climbing rings) as well as written psychoeducative information on the connection between physical activation and mood. Two, 5, and 7 weeks after the start, reminders were sent by e-mail or post. In addition, 5 weeks after the therapy began, all participants received additional psychoeducational information about goal setting according to the SMART method [23]. At the end of the 10-week intervention period, additional psychoeducational information on the implementation of exercise in everyday life was sent to the participants, and participants self-reported how many training units they had completed in a telephone conversation. For a more detailed description of the treatment, please see the study protocol [17].

### Recruitment and randomization

Patients were recruited between March 2017 and February 2018 by the study headquarters: Informational materials (e.g., posters, flyers) were distributed throughout the three study centers at locally based psychotherapists’ offices, primary care physicians’ offices, psychiatric hospitals, and other locations offering psychological services. Prior to each intervention wave, press releases were forwarded to several local radio stations and newspapers. In addition, presentations were held at local events (e.g., day of action against depression). Beyond these, a homepage (www.StudieKuS.de) and a Facebook account were created to inform about the process of the study. All people interested in the StudyKuS were invited to informational sessions by the study headquarters, which provided all relevant information about participating in the study (e.g., screening, randomization, and data acquisition).

Randomization was performed externally by the Institute of Medical Informatics, Biometrics, and Epidemiology (IMBE) of the Friedrich-Alexander University Erlangen-Nürnberg, only sharing screening date, participant code, sex, and severity of depression (categorized via PHQ-9 scores from screening: 8–14 mild to moderate, 15–19 moderately severe, 20–27 severe). Random allocation to the three groups (BPT, CBT, EP) was stratified by study center, and was performed blockwise by study wave, using a minimization algorithm to dynamically balance groups with respect to sex and disease severity. More specifically, at each wave, 1000 random permutations of group memberships for the new patients (with equally-sized groups) were generated, and the first permutation that minimizes the summed chi-squared statistics for the overall associations of group with sex and depression severity, respectively, was selected. The randomization algorithm was implemented in the statistical software environment R.

After randomization, the IMBE transmitted the final group allocation to the study headquartes, which then informed participants of their group assignment. Because of the obvious differences in therapy methods (climbing, CBT and exercise at home), participants and therapists could not be blinded to intervention, whereas data collectors were blinded (see data collection).

### Inclusion and exclusion criteria

Inclusion and exclusion criteria were assessed via self-report and verified with personal interviews by members of the study headquarters if there were any uncertainties. *Inclusion criteria* were depressive symptoms, operationalized by a PHQ-9 score of at least 8 points, in order to achieve a high sensitivity [24, 25], the possibility to get to the therapy locations, and informed consent. *Exclusion criteria* were: age < 18 years, body mass index (BMI) < 17.5 or > 40, simultaneous participation in psychotherapeutic group therapy, planned inpatient stay during the study period, psychotherapy or psychiatric medication started within the last 8 weeks, physical contraindication for bouldering (physical complaints or pregnancy), specific psychiatric disorders (psychosis or manic episode within the last 5 years, substance addiction with substance abuse or borderline personality disorder with self-harming behavior during the last year), and suicidality. For safety reasons, all participants signed an anti-suicide contract for the entire study period.

### Instruments and data collection

#### Instruments

*General Self-Efficacy Scale* (*GSE)* [26, 27] The GSE is a self-report questionnaire that consists of 10 items for assessing perceived self-efficacy (i.e., the main outcome of the present work) on a 4-point scale [26, 27]. A typical item is “Thanks to my resourcefulness, I can handle unforeseen situations.” Possible responses are 1 (*not at all true*), 2 (*hardly true*), 3 (*moderately true*), and 4 (*exactly true*), yielding a total score between 10 and 40 with higher scores indicating higher self-efficacy. In a recent representative German norm sample (*N* = 2,019), the mean value of the GSE score was 29.43 with a standard deviation of 5.36 [28]. Due to the norming by Hinz et al. [28], raw values of the GSE can be transformed into standardized T-scores with a mean of 50 and a standard deviation of 10, which allows an interpretation of the clinical significance of changes in the GSE: Because 68.2% of the norm sample have T-scores between 40 and 60 [28], T-Scores of < 40 can be interpreted as “below average”, whereas T-scores of > 60 can be considered “above average”. Regarding reliability, in a large sample of 19,120 people from 25 different countries [8] across all countries, a moderate to high internal consistency (Cronbach’s alpha = 0.86) was found. The large representative German norm sample (*N* = 2,019) even showed a high internal consistency with Cronbach’s alpha = 0.92 [28]. The construct validity of the GSE has been confirmed as several studies have shown significant positive correlations (convergent validity) with health behavior, well-being, active coping, resilience, optimism, and expected social support and significant negative correlations (divergent validity) with depression, anxiety, pain, and physical complaints [7, 8, 28]. Also the one-dimensional structure—and thus the factorial validity—has been validated in various studies by confirmatory factor analyses [8, 28].

*Montgomery–Åsberg Depression Rating Scale* (*MADRS)* [29] The MADRS is one of the most frequently used proxy-based instruments for assessing depression with a semistructured clinician-rated interview [30, 31]. The MADRS comprises 10 items, each of which is rated on a 7-point scale ranging from 0 to 6, resulting in a total value that ranges from 0 to 60. Higher scores indicate more severe depressive symptoms. To facilitate the collection of information necessary for a rating of the items, the *Structured Interview Guide for the Montgomery–Åsberg Depression Rating Scale* (*SIGMA)* was used. It shows good to excellent interrater reliability [32].

*The 9-item depression module of the Patient Health Questionnaire (PHQ-9)* [33, 34] The PHQ-9 is a short self-report questionnaire often used in primary care settings for the screening of depression [35]. Each of the 9 items is rated with respect to experiences during the last 2 weeks on a 4-point unipolar scale (0 = *not at all*; 1 = *several days*; 2 = *more than half the days*; 3 = *nearly every day*), resulting in scores ranging from 0 to 27. Higher scores indicate more severe depressive symptoms.

Beyond that, sociodemographic data, BMI, and current therapeutic treatment (psychiatric medication, additional psychotherapy) were assessed via self-report. A full description of all outcomes assessed in the StudyKuS can be found in the study protocol [17].

#### Data collection

Clinical psychology students were trained at the study headquarters to collect data using computer-assisted telephone interviews (CATIs). Data were collected at baseline (t0), directly after the 10-week intervention period (t1), as well as 3 months (t2), 6 months (t3), and 12 months (t4) after the end of the intervention period. Because we are investigating three different non-pharmacological interventions, neither the participants nor the people involved in conducting the interventions could be blinded to group allocation. Nevertheless, all students conducting CATIs were blinded to participants’ group allocation. Additionally, before each interview, participants were informed that their group allocation was confidential and that they should not disclose information about which group they had been allocated to.

### Statistical analysis

All participants in each group (BPT, CBT, and EP) were combined across the three study centers and all waves for the main analyses to increase the statistical power. Descriptive statistics (frequencies, means, and standard deviations) were computed to demonstrate sample and baseline characteristics. In addition, to assess the quality of the randomization, differences between the three groups in the variables of interest were evaluated by computing univariate analyses of variance (ANOVAs) and chi-square ($\chi$^*2*^) tests. The underlying assumptions of parametric tests were checked with the Kolmogorov–Smirnov test and Levene’s test. A missing data evaluation was carried out, and missing values were imputed by applying the expectation maximization (EM) algorithm. All data were checked for plausibility. The primary data analysis strategy was intention to treat (ITT). Participants who dropped out during the intervention period were subsequently interviewed and included in the ITT analyses. Dropout analyses were calculated to check for differences between participants who dropped out and those who completed the study, using χ^2^ tests and *t* tests for independent samples.

To check for pre-post changes within the groups in the main outcome criterion of the present work (i.e., perceived self-efficacy assessed with the GSE), paired *t* tests were computed to compare the changes between t0 and t1 in all three groups. To compare the effect of BPT on perceived self-efficacy in people with depression with the effect of physical activity alone in the form of the EP, a mixed ANOVA with the within-subject variable time (two-fold: t0 and t1) and the between-subject variable group (two-fold: BPT and EP) was carried out. For this purpose, only participants in the BPT and EP groups (ITT: *n* = 156; PP: *n* = 133) were included. To avoid the overinterpretation of “unadjusted” effects, we decided a priori to carry out a hierarchical multiple regression analysis to control for other potential predictors of the dependent variable GSE. Thus, a hierarchical regression analysis was calculated to predict the postintervention (t1) GSE score from group allocation (BPT vs. EP), controlling for the GSE score at baseline (GSE t0) as well as for demographic variables (age, sex), other therapeutic treatments (antidepressant medication, additional psychotherapy), and severity of depression at baseline (MADRS t0). All blocks of the hierarchical regression analysis were added using the “enter” method. Collinearity statistics were examined in advance. There were no issues with multicollinearity.

**Fig. 1:**
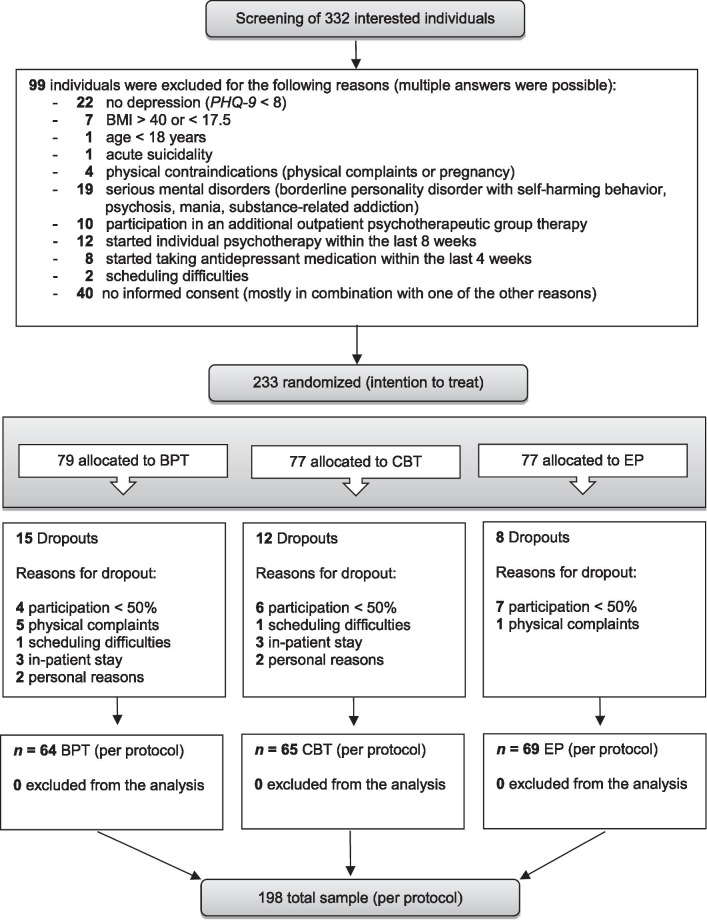
CONSORT Flow Chart for the StudyKuS. *Note:* First published in Karg N, Dorscht L, Kornhuber J, Luttenberger K. Bouldering psychotherapy is more effective in the treatment of depression than physical exercise alone: results of a multicentre randomised controlled intervention study. BMC Psychiatry. 2020;20:116. https://doi.org/10.1186/s12888-020-02518-y. Copyright 2020 by the authors, licensed under a CC BY International License. BPT: Bouldering psychotherapy, CBT: Cognitive behavioral group therapy, EP: Home-based physical exercise program

As a sensitivity analysis, additional analyses with the per protocol (PP) sample (see Fig. 1), were computed and compared with the results of the ITT analyses. Furthermore, a mixed ANOVA with the within-subject variable time (two-fold: t0 and t1) and the two between-subject variables group (two-fold: BPT and EP) and region (three-fold: Erlangen/Nürnberg/Fürth, Weyarn, and Berlin) was computed to conduct an exploratory evaluation of possible study center effects.

To investigate whether the effect of BPT on self-efficacy differed significantly from that of CBT, an additional mixed ANOVA with the within-subject variable time (two-fold: t0 and t1) and the between-subject variable group (two-fold: BPT and CBT) was conducted. For this purpose, only participants in the BPT and CBT groups (ITT: *n* = 156; PP: *n* = 134) were included. As a sensitivity analysis, additional analyses with PP data were computed and compared with the results of the ITT analyses.

Furthermore, mediation analyses were performed using the PROCESS macro by Andrew F. Hayes [36]. Three separate mediation analyses for each group (BPT, CBT, EP) were computed in the PP sample to analyze whether depression at baseline (MADRS t0) would predict the postintervention depression score (MADRS t1) and whether the direct path would be mediated by perceived self-efficacy (GSE t1) while controlling for self-efficacy before therapy (GSE t0).

As a measure of effect size, Cohen’s *d* was calculated. A type I error rate (alpha) of less than 5% was considered indicative of statistical significance. However, because we performed two main analyses (i.e., BPT vs. EP and BPT vs. CBT) in one sample, we had to adjust for multiple testing. Therefore, after we applied the Bonferroni correction (alpha/number of analyses) [37], the significance of all outcome analyses was indicated at a type I error rate (alpha) of less than 2.5%. Statistical analyses were computed with the IBM SPSS Statistics 21 and 24 software.

## Results

### Description of study participants

The intervention period ran from May 2017 to June 2018 in consecutive waves. Of 332 individuals who attended the screening, 99 did not meet the inclusion criteria (see Fig. 1 for the reasons for exclusion). A total of 233 participants were included in the study (ITT sample) and randomly assigned to the BPT group (*n* = 79), EP group (*n* = 77), and CBT group (*n* = 77). Figure 1 shows the study’s CONSORT Flow Chart, which was already published by Karg et al. [18]. During the intervention period, 35 participants dropped out of the study (for the reasons, see Fig. 1).

The baseline characteristics of the ITT sample and group differences between the BPT, EP, and CBT groups are shown in Table 1.

**Table 1:** Baseline characteristics of the intention to treat sample

Variable	BPT (*n* = 79)		EP (*n* = 77)		CBT (*n* = 77)		Total (*n* = 233)		Test of group differences		
									*F*^a^	χ^2 b^	*p*
Age ^a^, *M* (*SD*)	41.75	(12.66)	42.68	(12.52)	40.25	(11.35)	41.56	(12.18)	0.78		.461
Sex: female, *n* (%)	54	(68,4)	52	(67.5)	51	(66.2)	157	(67.4)		0.08	.960
BMI, *M* (*SD*)	23.92	(3.37)	24.98	(4.33)	24.57	(4.17)	24.49	(3.98)	1.41		.245
School education, *n* (%)										5.12	.529
< 9 years	0	(0.0)	1	(1.3)	0	(0.0)	1	(0.4)			
9 years	9	(11.4)	5	(6.5)	9	(11.7)	23	(9.9)			
10 years	18	(22.8)	24	(31.2)	17	(22.1)	59	(25.3)			
≥ 12 years	52	(65.8)	47	(61.0)	51	(66.2)	150	(64.4)			
Employed: yes, *n* (%)	50	(63.3)	42	(54.5)	45	(58.4)	137	(58.8)		3.44	.488
Additional psychotherapy: yes, *n* (%)	42	(53.2)	27	(35.1)	38	(49.4)	107	(45.9)		5.69	.058
Antidepressants: yes, *n* (%)	41	(51.9)	37	(48.1)	39	(50.6)	117	(50.2)		0.24	.887
Depression at baseline (t0)											
MADRS, *M (SD)*	23.46	(8.93)	22.27	(9.12)	24.04	(7.69)	23.26	(8.60)	0.43		.432
PHQ-9, *M (SD)*	13.66	(5.49)	13.36	(5.15)	13.81	(4.65)	13.61	(5.09)	0.15		.861
Study center, *n* (%)										10.99	.027*
Erlangen/Nürnberg/Fürth	39	(49.4)	52	(67.5)	37	(48.1)	128	(54.9)			
Weyarn/Munich	20	(25.3)	12	(15.6)	27	(35.1)	59	(25.3)			
Berlin	20	(25.3)	13	(16.9)	13	(16.9)	46	(19.7)			

BMI = Body Mass Index; MADRS = Montgomery and Åsberg Depression Rating Scale, higher scores indicate more severe depressive symptoms, range: 0–60; PHQ-9 = The 9-item depression module of the Patient Health Questionnaire, higher scores indicate more depressive symptoms, range: 0–27

*p < .05, significant* p*-values are printed in bold; n = 233 (intention to treat)

^a^*F* value from an univariate ANOVA with the between-subject factor group (BPT, EP, CBT)

^b^Chi-square (χ^2^) value from a chi-square test

The overall mean age in the ITT sample was 41.56 years (*SD* = 12.18; Range: 18 to 70), and the mean BMI was 24.49 (*SD* = 3.98; Range: 17.57 to 39.45). The mean MADRS score was 23.26 (*SD* = 8.60; Range: 0 to 46), and the mean PHQ-9 score was 13.61 (*SD* = 5.09; Range: 0 to 25), which indicates moderate depression [24, 32, 33, 38]. About two thirds of the participants were women (67.4%) and employed (58.8%). The majority had completed 12 years of schooling (64.4%). Nearly half of all participants also received additional psychotherapy (45.9%) or took antidepressants (50.2%). Most participants were recruited in Erlangen/Nürnberg/Fürth (54.9%), followed by Munich/Weyarn (25.3%) and Berlin (19.7%).

In addition, Table 1 shows that the different groups (BPT, CBT, and EP) in the ITT sample did not differ significantly from one another in age, BMI, or depression, which could be statistically confirmed by one-factor ANOVAs. The chi-square tests also did not show any significant differences in education, employment, the use of antidepressants, or participation in additional psychotherapy. However, the participants were not evenly distributed across the groups at each study center, as Table 1 illustrates, χ^2^(4) = 13.87, *p* = 0.008.

The sample characteristics and differences found in the ITT sample were similar to the ones found in the PP sample except that the groups in the PP sample differed significantly on the additional psychotherapy variable: People in the EP group participated in additional psychotherapy less frequently (34.8%) than people in the BPT (54.7%) or CBT (52.3%), χ^2^(2) = 6.39, *p* = 0.041.

Participants who dropped out between t0 and t1 (*n* = 35) did not differ from the remaining PP sample in age, sex, BMI, employment status, or additional psychotherapy or antidepressants received. However, they differed in self-rated depression (PHQ-9 scores at t0; dropouts: *M* = 15.54 vs. PP: *M* = 13.27; *t*(231) = 2.46, *p* = 0.014), though they did not differ in terms of proxy-based clinician-rated depression (MADRS scores at t0; dropouts: *M* = 24.89 vs. PP: *M* = 22.97; *t*(231) = 1.22, *p* = 0.225).

### Descriptive analyses

The mean values, standard deviations, and standardized T-scores of the overall ITT sample (*n* = 233) on the GSE at t0 and t1 are illustrated in Table 2. In all three groups, perceived self-efficacy improved significantly, although the observed changes were highest in the BPT group with 3.04 GSE points, *t*(78) = 5.59, *p* < 0.001, followed by 2.44 points in the CBT group, *t*(76) = 4.96, *p* < 0.001, and 1.26 points in the EP group, *t*(76) = 2.71, *p* = 0.008. The descriptively observed differences between the BPT, CBT, and EP groups on the GSE score at baseline (t0) were not significant according to a one-factor ANOVA, *F*(2, 230) = 1.37, *p* = 0.257, partial η^2^ = 0.01.

**Table 2:** Means, standard deviations, and T-scores for the GSE at t0 and t1 in the intention to treat sample

	BPT (*n* = 79)		EP (*n* = 77)		CBT (*n* = 77)		Total sample (*n* = 233)	
	*M* (*SD*)	T-score	*M* (*SD*)	T-score	*M* (*SD*)	T-score	*M* (*SD*)	T-score
t0	21.96 (4.55)	36	22.17 (4.76)	36	23.17 (5.25)	38	22.43 (4.87)	37
t1	25.00 (5.36)	42	23.43 (5.42)	39	25.61 (4.61)	43	24.68 (5.20)	41

T-scores according to the representative German norm sample by Hinz et al. (2006), < 40: “below average”, 40–60: “normal”, > 60: “above average”; GSE: General Self-Efficacy Scale, higher scores indicate higher perceived self-efficacy, range: 10–40; t0: baseline data collection, t1: data collection directly after the ten-week intervention period

At baseline (t0), BPT participants were about 1.5 *SD* units below the mean of the norm sample by Hinz et al. [28]. At the end of the intervention period (t1), the mean GSE score in the BPT group increased by 0.60 *SD* units to a T-score of 42, which is considered “average” or healthy, because 68.2% of the norm sample have T-scores between 40 and 60. The development in the CBT group was quite similar (an increase of 0.50 *SD* units to a T-score of 43), whereas the GSE score of participants in the EP increased by only 0.30 *SD* units to a T-score of 39, which is considered “below average” [28].

Kolmogorov–Smirnov tests as well as Q-Q plots and Histogramms showed that the dependent variable GSE was not normally distributed at t0, but normally distributed at t1 in each group in the ITT sample. Levene tests showed that the assumptions of homogeneity of variance could be confirmed. In addition, the assumption of sphericity is always given in a mixed ANOVA with a two-fold within-subject factor [39]. Therefore, ANOVAs and *t* tests were computed because the various subgroups in the present overall sample had a sample size of *n* > 30 [39] and ANOVAs and *t* tests are considered to be very robust against violations of assumptions of normality, especially, if no other assumptions are violated [39, 40].

### BPT versus EP

#### ANOVA

**Fig. 2:**
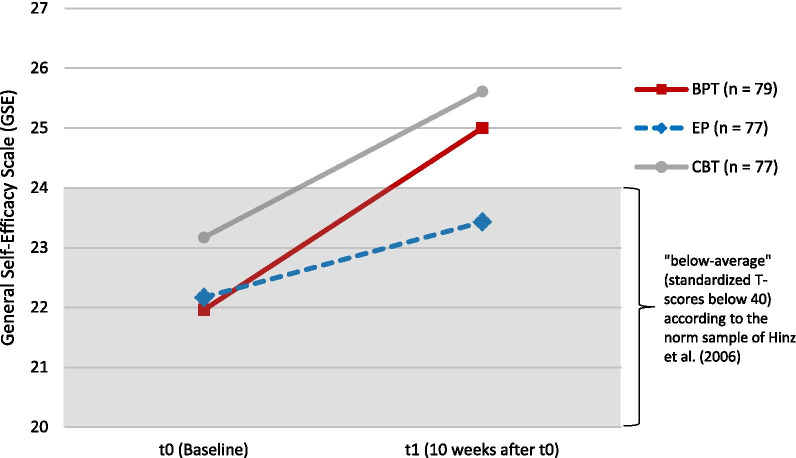
Change in perceived self-efficacy between t0 and t1 on the GSE in the intention to treat sample. *Note:* BPT: Bouldering psychotherapy, CBT: Cognitive behavioral group therapy, EP: Home-based physical exercise program, GSE: General Self-Efficacy Scale, higher scores indicate higher perceived self-efficacy, range: 10–40; t0: baseline data collection, t1: data collection directly after the ten-week intervention period; *n* = 233 (intention to treat)

A mixed ANOVA in the ITT sample with the dependent variable GSE, the within-subject variable time (two-fold: t0 and t1) and the between-subject variable group (two-fold: BPT and EP) showed no significant main effect of group, *F*(1, 154) = 0.88, *p* = 0.349, partial η^2^ = 0.01. However, a significant main effect of time was found, which means that perceived self-efficacy showed a substantial increase over time in both groups (BPT and EP), *F*(1, 154) = 35.20, *p* < 0.001, partial η^2^ = 0.19. Beyond this, a significant interaction between group and time was found. This means that the strength of the increase over time depended on the assignment to either the BPT or EP group, *F*(1, 154) = 5.95, *p* = 0.016, partial η^2^ = 0.04. Accordingly, Fig. 2 shows that perceived self-efficacy showed more improvement from t0 to t1 in the BPT than in the EP group. The effect size for this observed effect was Cohen's *d* = 0.39 (95% CI [0.07, 0.71]), which is considered a moderate effect according to Cohen [41].

The PP analysis showed comparable results: There was no significant main effect of group, *F*(1, 131) = 0.18, *p* = 0.675, partial η^2^ = 0.00. As in the ITT analysis, a significant main effect of time was found. This main effect of time means that perceived self-efficacy showed a substantial increase over time in both groups (BPT and EP), *F*(1, 131) = 36.88, *p* < 0.001, partial η^2^ = 0.22. In addition, as in the ITT analysis, a significant interaction between group and time was found. This interaction means that the strength of the increase over time depended on group assignment, *F*(1, 131) = 7.34, *p* = 0.008, partial η^2^ = 0.05. The effect size for this observed effect was also moderate, Cohen's *d* = 0.47 (95% CI [0.12, 0.81]).

#### Hierarchical multiple regression analysis

To avoid the overinterpretation of “unadjusted” effects, a confounder-adjusted hierarchical regression analysis was calculated with the ITT sample (*n* = 233) to predict the postintervention (t1) GSE score from group allocation (BPT vs. EP), controlling for the GSE score at baseline (t0) and for demographic variables (age, sex), other therapeutic treatments (antidepressant medication, additional psychotherapy), and severity of depression at baseline (MADRS t0). An overview of the regression analysis is presented in Table 3.

**Table 3:** Hierarchical multiple regression analysis for variables predicting perceived self-efficacy (GSE score) at t1 in the intention to treat sample

		*B*	*SE(B)*		β		*p*		Δ*R*^*2*^
*Step 1*									.352, *p* < .001***
	GSE sum score (t0)	0.69	0.08		.59		< .001***		
*Step 2*									.016, *p* = .584
	GSE sum score (t0)	0.66	0.08		.56		< .001***		
	MADRS sum score (t0)	– 0.05	0.04		– .08		.254		
	Age	0.02	0.03		.04		.570		
	Sex: female	1.02	0.79		.09		.199		
	Psychotherapy: yes	0.57	0.74		.05		.444		
	Antidepressants: yes	– 0.48	0.73		– .04		.515		
*Step 3*									.025**,** *p* = .014*
	GSE sum score (t0)	0.66	0.08		.56		< .001***		
	MADRS sum score (t0)	– 0.06	0.04		– .10		.166		
	Age	0.02	0.03		.05		.471		
	Sex: female	1.05	0.78		.09		.179		
	Psychotherapy: yes	0.21	0.74		.02		.773		
	Antidepressants: yes	– 0.46	0.72		– .04		.527		
	Group allocation: BPT	1.76	0.70		.16		.014*		
Total *R*^*2*^									.393, *p* < .001***

MADRS: Montgomery–Åsberg Depression Rating Scale, higher scores indicate more severe symptoms, range: 0–60; GSE: General Self-Efficacy Scale, higher scores indicate higher perceived self-efficacy, range: 10–40; Sex: 1 (female), 0 (male); Psychotherapy: 1 (yes), 0 (no); Antidepressants: 1 (yes), 0 (no); Group allocation: 1 (BPT), 0 (EP); t0: baseline data collection, t1: data collection directly after the ten-week intervention period

*p < .05, ***p < .001, significant* p*-values are printed in bold; *n* = 156 (intention to treat, BPT & EP)

In the first step of the hierarchical regression, the GSE score at t0 was added to the regression model. The GSE score at t0 emerged as a significant predictor (β = 0.69, *p* < 0.001) and explained 35.2% of the variance. In the second step, the remaining control variables (age, sex, antidepressant medication, additional psychotherapy) were added to the regression model, whereby none of the newly added variables represented a significant predictor because only an additional 1.6% of the variance could be explained. The change in *R*^*2*^ was not significant. Adding group allocation to the regression model in the third step revealed a significant effect of group allocation (BPT vs. EP) on the postintervention GSE score at t1, with BPT participants showing significantly higher perceived self-efficacy than participants in the EP group (β = 0.16, *p* = 0.014). This third step explained an additional 2.5% of the variance, and the change in *R*^*2*^ was significant. The final overall model explained 39.3% of the variance and was significant, *F*(7, 148) = 13,69, *p* < 0.001.

The PP analysis showed comparable results: Only the GSE score at t0 (β = 0.59, *p* < 0.001) and group allocation (β = 0.18, *p* = 0.013) emerged as significant predictors. The final overall model explained 38.6% of the variance and was significant, *F*(7, 125) = 11.24, *p* < 0.001.

#### Study center effects

A mixed ANOVA in the ITT sample with the within-subject variable time (two-fold: t0 and t1) and the two between-subject variables—group (two-fold: BPT and EP) and region (three-fold: Erlangen/Nürnberg/Fürth, Weyarn, and Berlin)—was conducted to evaluate possible study center effects. In line with the previous results, there was no significant main effect of group, *F*(1, 150) = 0.07, *p* = 0.794, partial η^2^ = 0.00, but there was a significant main effect of time, *F*(1, 150) = 23.83, *p* < 0.001, partial η^2^ = 0.14, and a significant interaction between group and time *F*(1, 150) = 5.94, *p* = 0.016, partial η^2^ = 0.04. However, there was no significant interaction between time and study center, which means that no difference in the significant main effect of time (i.e., the significant GSE score increase from t0 to t1 in both the BPT and EP groups), could be identified between the different study centers, *F*(2, 150) = 1.02, *p* = 0.363, partial η^2 ^= 0.01. In addition, there was no significant interaction between group, time, and study center, meaning that no difference was found between the study centers regarding the significant interaction between group and time, *F*(2, 150) = 0.39, *p* = 0.681, partial η^2^ = 0.01. Furthermore, there was also no significant main effect of study center, *F*(2, 150) = 0.24, *p* = 0.785, partial η^2^ = 0.00, and no significant interaction between study center and group, *F*(2, 150) = 0.72, *p* = 0.487, partial η^2^ = 0.01.

The PP analysis showed comparable results. A significant main effect of time, *F*(1, 127) = 24.16, *p* < 0.001, partial η^2^ = 0.16, and a significant interaction between group and time, *F*(1, 127) = 6.82, *p* = 0.010, partial η^2^ = 0.05, were found. But there was no significant main effect of group, *F*(1, 127) = 0.00, *p* = 0.976, partial η^2^ = 0.00, no significant main effect of study center, *F*(2, 127) = 0.29, *p* = 0.747, partial η^2^ = 0.01, no significant interaction between group, time, and study center, *F*(2, 127) = 0.17, *p* = 0.847, partial η^2^ = 0.00, no significant interaction between study center and group, *F*(2, 127) = 0.31, *p* = 0.737, partial η^2^ = 0.01, and no significant interaction between time and study center, *F*(2, 127) = 1.08, *p* = 0.343, partial η^2^ = 0.02.

### BPT versus CBT

A mixed ANOVA in the ITT sample with the within-subject variable time (two-fold: t0 and t1) and the between-subject variable group (two-fold: BPT and CBT) showed no significant main effect of group, *F*(1, 154) = 1.69, *p* = 0.196, partial η^2^ = 0.01. Furthermore, no significant interaction between group and time, *F*(1, 154) = 0.64, *p* = 0.425, partial η^2^ = 0.00, was found, which means that the strength of the increase over time was independent of group allocation to either the BPT or CBT group. Accordingly, Fig. 2 shows that the improvement in perceived self-efficacy was comparable from t0 to t1 in the BPT and CBT groups. However, a significant main effect of time was found, which means that perceived self-efficacy showed a substantial increase over time in both the BPT and CBT groups, *F*(1, 154) = 54.66, *p* < 0.001, partial η^2^ = 0.26.

The PP analysis showed comparable results: Neither a significant main effect of group, *F*(1, 127) = 3.67, *p* = 0.058, partial η^2^ = 0.03, nor a significant interaction between group and time, *F*(1, 127) = 1.68, *p* = 0.198, partial η^2^ = 0.01, was found. As in the ITT analysis, a significant main effect of time was found, *F*(1, 127) = 48.49, *p* < 0.001, partial η^2^ = 0.28.

Due to the nonsignificant results in the ANOVA regarding differences between BPT and CBT, no further adjusted analyses were conducted.

### Mediation analyses

Three separate mediation analyses for each group (BPT, CBT, EP) were computed in the PP sample. In all three groups, a total effect of depression score at baseline (MADRS t0) on the postintervention depression score (MADRS t1) was observed (BPT: *B* = 0.32, *p* = 0.028; CBT: *B* = 0.78, *p* < 0.001; EP: *B* = 0.56, *p* = 0.005). After entering the mediator into the model, we found that the relationship between depression at baseline and postintervention was partially mediated (23%) by perceived self-efficacy only in the CBT group (direct effect: *B* = 0.60, *p* < 0.001; indirect effect: *B* = 0.18, 95% CI [0.02, 0.38]). A significant mediation effect was not found in the BPT group or in the EP group.

## Discussion

The StudyKuS is the first randomized controlled trial to compare the positive effect of therapeutic climbing on perceived self-efficacy in people with depression with that of a home-based physical exercise program and state-of-the-art CBT. In all groups, preintervention perceived self-efficacy could be rated “below average” [28]. Perceived self-efficacy was effectively enhanced in all three groups, whereas only BPT and CBT participants’ postintervention self-efficacy could be rated “normal” or healthy [28]. In comparison with the EP group, BPT participants had a significantly greater increase in self-efficacy, whereas we could not find any differences between the BPT and CBT groups. With an effect size (Cohen’s *d*) of 0.39, which can be considered moderate according to Cohen [41], the effect of BPT compared with the EP on perceived self-efficacy can be considered clinically relevant.

Our results are in accordance with previous findings on the positive effects of physical exercise on self-efficacy in a review by McAuley and Blissmer [42] and of group psychotherapy on self-efficacy in a randomized controlled trial by Guo et al. [43]. Furthermore, our results are also consistent with findings on the positive effect of therapeutic climbing on self-efficacy in our pilot study [16] and in two reviews [14, 15]. Beyond the previous results, the present work was able to show that the effect of therapeutic climbing on perceived self-efficacy in people with depression is superior to that of physical activity alone.

Furthermore, the results indicate that the effect of BPT on depression previously found by Karg et al. [18] was not mediated by perceived self-efficacy. A mediation effect was only found in the CBT group, which is in line with literature that considers perceived self-efficacy to be a common factor of psychotherapy [4, 5]. Thus, the effect of BPT on depression [18] does not seem to depend on an increase in self-efficacy. It seems as though, in CBT, the effect on depression severity is at least partially due to an increase in self-efficacy, whereas in BPT, other—currently unknown—factors are relevant. Future studies should focus on the modes of action of BPT to gain better insight into how BPT unfolds its effect on depression severity. Therefore, it would be beneficial to compare BPT with non-therapeutic bouldering as well as other physical activities in a group setting.

### Implications

Nevertheless, the results of the current study indicate that therapeutic climbing in the form of BPT leads to a significant and clinically relevant improvement in perceived self-efficacy in people with depression and that this effect is superior to that of physical activity alone in the form of the EP and comparable to that of CBT. BPT improved the protective and resilience-related factor of perceived self-efficacy in people with depression, which means that the resistance to potential environmental stressors (i.e., resilience) was strengthened. Thus, the risk of chronification of the depressive symptoms or development of another comorbid mental disorder may be reduced in accordance with the vulnerability stress model by Wittchen and Hoyer [44]. These results also give reason to expect that BPT could be suitable in the area of prevention. However, it would also be conceivable that the positive effect of BPT on people with depression can also be transferred to other health problems in which self-efficacy plays a role. For example, people suffering from chronic pain may benefit from BPT, as Luszczynska et al. [7] were able to show in a survey of 1,933 people in Germany, Poland, and South Korea that a higher level of perceived self-efficacy is also associated with a higher pain tolerance. This idea has been supported by initial findings indicating positive effects of therapeutic climbing on chronic back pain [45–47]. Furthermore, anxious people or people with an anxiety disorder may also benefit from a BPT intervention, as Scholz et al. [8] were able to show in a survey of 19,120 people in 25 countries that high perceived self-efficacy is associated with lower anxiety. Nevertheless, further research investigating the effects of therapeutic climbing on perceived self-efficacy for other mental or physical disorders (e.g., anxiety disorders or chronic pain) as well as in the field of health prevention is needed to support these assumptions.

Beyond the findings discussed above, no differences were found between BPT and CBT with respect to positive effects on self-efficacy, which suggests that BPT should be used in health care besides state-of-the-art CBT. Participation in a conventional CBT may be a deterrent for some people because CBT is strongly associated with the treatment of mental illness and therefore with the stigma associated with mental illness in our society [48]. On the other hand, the main focus of BPT is initially on bouldering and thus on a sport that is becoming increasingly popular [9, 10]. Thus, the access to BPT could be considered more “low-threshold”. Furthermore, the use of BPT as a complementary therapeutic procedure within a multimodal in-patient treatment program in psychiatric, psychotherapeutic or psychosomatic clinics seems promising. BPT could make a major contribution to the effectiveness of the entire multimodal inpatient treatment program due to its clinically relevant positive effect on perceived self-efficacy, an important common factor of psychotherapy [4, 5].

### Strengths

The recruitment of participants in three different regions in Germany and the selection of only few inclusion and exclusion criteria maximized the external validity as well as the generalizability of the findings. Randomized allocation to BPT, CBT, or EP ensured the control of relevant confounding factors and a high level of internal validity. Perceived self-efficacy was assessed with the GSE, a widely used and validated self-report questionnaire. Furthermore, our study is the first randomized controlled trial to compare BPT to physical exercise alone and state-of-the-art CBT regarding the effect on perceived self-efficacy.

### Limitations

Nevertheless, “absence of evidence is not evidence of absence” [49]. Therefore, noninferiority trials should be conducted in the future [50, 51] in order to support the first indications of noninferiority between BPT and CBT with respect to the effect on perceived self-efficacy that can be drawn from the present study. Additionally, the GSE was only a secondary outcome of the StudyKuS, and several aspects of the study (e.g., sample size estimation) were therefore not centered around the GSE (for more information, please see our study protocol [17]). However, between-group comparisons at least revealed no significant differences in perceived self-efficacy between all three groups (BPT, CBT, EP) at baseline.

Despite randomization, groups in the PP sample differed significant at baseline: people in the EP participated less frequently in additional psychotherapy than those in the BPT or CBT group. However, the confounder-adjusted hierarchical multiple regression analysis showed that participation in additional psychotherapy at baseline had no significant effect on the postintervention GSE score. Beyond this finding, participants were not evenly distributed among the groups within each study center at baseline. Thus, an additional mixed ANOVA controlling for possible study center effects was conducted, whereby no differences between the study centers regarding the effect on perceived self-efficacy were found.

Regarding the setting, it should be taken into account that BPT and CBT were conducted as a group intervention. Therefore, a great deal of vicarious experience regarding novel and difficult situations could be gained in BPT and CBT, whereas the EP was carried out alone at home without the possibility of having vicarious experiences. Because vicarious experience is one major source of perceived self-efficacy [1, 3], it is not possible to rule out the effect of group (i.e., vicarious experience) when comparing BPT and CBT with the EP.

Another limitation of the study is the fact that interested individuals with depression had to request participation on their own and come to an informational session before they could participate in the study. Thus, the barrier to participation may have been too high for people with pronounced lassitude or less motivation.

The present work can only draw conclusions about the immediate effects of therapeutic climbing on perceived self-efficacy in people with depression. Possible long-term effects have to be investigated by analyzing follow-up data, which will be done by our work group in future work.

## Conclusions

The results of the current study show that BPT with a scope of 10 h in a group setting can contribute to a clinically relevant improvement of perceived self-efficacy in people with depression. In this context, the effect of BPT is superior to that of physical activity alone in form of the EP. The results also provide initial indications that the effect of BPT on self-efficacy is comparable to that of state-of-the-art CBT, suggesting a strong case for a broader use of BPT as a supplement to existing health care services, especially when perceived self-efficacy should be enhanced. Future studies should focus on the modes of action of BPT and its effect on perceived self-efficacy in people with other mental or physical disorders for a more specific implementation of BPT in clinical practice.

## Data Availability

All the results supporting our conclusions are contained in the manuscript. The data sets that were used and/or analyzed in the current study are available from the corresponding author upon reasonable request after the publication of the results.

## Abbreviations

BPT: Bouldering Psychotherapy
BMI: Body Mass Index
CATI: Computer-Assisted Telephone Interview
CBT: Cognitive Behavioral Group Therapy
CI: Confidence Interval
EP: Home-Based Physical Exercise Program
GSE: General Self-Efficacy Scale
ITT: Intention to treat
MADRS: Montgomery–Åsberg Depression Rating Scale
M: Mean
PHQ-9: The 9-item depression module of the Patient Health Questionnaire
PP: Per protocol
SD: Standard Deviation
